# Bone marrow stromal cells interaction with titanium; Effects of composition and surface modification

**DOI:** 10.1371/journal.pone.0216087

**Published:** 2019-05-22

**Authors:** Murali Krishna Duvvuru, Weiguo Han, Prantik Roy Chowdhury, Sahar Vahabzadeh, Federico Sciammarella, Sherine F. Elsawa

**Affiliations:** 1 Department of Mechanical Engineering, Northern Illinois University, Dekalb, Illinois, United States of America; 2 Department of Molecular, Cellular and Biomedical Sciences, University of New Hampshire, Durham, New Hampshire, United States of America; Charles P. Darby Children’s Research Institute, UNITED STATES

## Abstract

Inflammation and implant loosening are major concerns when using titanium implants for hard tissue engineering applications. Surface modification is one of the promising tools to enhance tissue-material integration in metallic implants. Here, we used anodization technique to modify the surface of commercially pure titanium (CP-Ti) and titanium alloy (Ti-6Al-4V) samples. Our results show that electrolyte composition, anodization time and voltage dictated the formation of well-organized nanotubes. Although electrolyte containing HF in water resulted in nanotube formation on Ti, the presence of NH4F and ethylene glycol was necessary for successful nanotube formation on Ti-6Al-4V. Upon examination of the interaction of bone marrow stromal cells (BMSCs) with the modified samples, we found that Ti-6Al-4V without nanotubes induced cell proliferation and cluster of differentiation 40 ligand (CD40L) expression which facilitates B-cell activation to promote early bone healing. However, the expression of glioma associated protein 2 (GLI2), which regulates CD40L, was reduced in Ti-6Al-4V and the presence of nanotubes further reduced its expression. The inflammatory cytokine interleukin-6 (IL-6) expression was reduced by nanotube presence on Ti. These results suggest that Ti-6Al-4V with nanotubes may be suitable implants because they have no effect on BMSC growth and inflammation.

## Introduction

Commercially pure titanium (CP-Ti) and titanium alloy (Ti-6Al-4V) are widely used as dental and orthopedic implants due to their biocompatibility, excellent corrosion resistance and desired mechanical properties. This includes properties such as low Young’s modulus, low density and fatigue resistance [1–3]. However, the formation of a fibrous capsule around titanium implants and the resultant implant loosening can cause severe pain for patients, which often requires revision surgery [4]. Surface modification techniques such as applying an osteoconductive coating, alkali treatment, acidic treatment, and electrochemical anodization are promising tools to enhance osseointegration of Ti implants [5]. Among the various techniques, introducing TiO_2_ nanotubes on the surface of Ti through anodization has gained attention as it is a simple, cost efficient and well controlled methodology. However, parameters such as electrolyte composition, voltage and the duration of anodization have been shown to alter the morphology of the surface [6,7]. In the current study, we used five different conditions to investigate and compare the effects of substrate composition and anodization parameters on successful formation of nanotubes on both CP-Ti and Ti-6Al-4V [8,9]. We hypothesized that different conditions are needed to achieve well-formed nanotubes on CP-Ti and Ti-6Al-4V.

Several studies reported the efficiency of TiO_2_ nanotubular layer on *in vitro* cell adhesion and proliferation, protein adsorption, and on *in vivo* osseointegration [10–13]. The presence of nanotubes not only enhances the surface roughness and hydrophilicity, but also activates angiogenic factors [12]. In addition, nanotube incorporation increases *in vivo* collagen and osteocalcin expression, and bone-implant interfacial strength which further improves osseointegration [13]. However, the interaction between nanotubes and bone marrow stromal cells (BMSCs) is less understood and the resultant inflammatory response has not been reported. BMSCs play an important role in blood and stem cell development and differentiation [14,15]. These cells can also differentiate into osteoblasts or adipocytes under the proper cell culture conditions [16], or depending on the type of biomaterial they are cultured on [17]. Therefore, understanding the nature of the interaction between BMSCs and titanium as a function of composition and surface roughness is necessary to predict blood cell development and bone healing.

In this study, we investigated the effects of substrate composition, electrolyte, voltage and anodization time on successful nanotube arrangement and studied the effects of titanium composition and nanotube presence on their interaction with human BMSCs. Nanotube microstructures were evaluated using scanning electron microscopy (SEM) and well-formed structures were used to investigate early proliferation of BMSCs and their inflammatory responses.

## Materials and methods

### Sample preparation

Grade 2 CP-Ti sheets were purchased from President Titanium, MA, USA. Ti-6Al-4V cylinders were formed using an additive manufacturing technique (LENS) with laser power of 645 W and travel speed of 60 inch/min. These cylinders were then machined and cut into Ti-6Al-4V discs. CP-Ti and Ti-6Al-4V substrates with a diameter of 11 mm and a thickness of 2 mm were grinded using silicon carbide papers from 320 to 800 grit, followed by polishing using a MasterTex polishing cloth. Samples were then cleaned in DI water and acetone, and then air dried. The titanium anode and a platinum foil cathode (Alfa Aesar, Tewksbury, MA,USA) were connected to a DC power supply (Agilent E3612A) and suspended in a beaker containing the electrolyte with different compositions as indicated in Table 1. After anodization, the samples were rinsed with DI water and then air dried. The conditions outlined below were selected as previously described [8,9].

**Table 1:** Anodization process conditions.

Condition	Electrolyte	Voltage (V)	Time (min)
**A**	1 vol. % HF in DI water	20	45
**B**	1 vol. % HF in DI water	20	60
**C**	1 vol. % HF in DI water	30	60
**D**	1 vol. % HF, 0.5 wt. % NH_4_F, 10 vol. % DI water in Ethylene Glycol medium	40	60
**E**	0.25 wt. % NH_4_F, 2 vol. % DI Water in Ethylene Glycol Medium	30	180

### Characterization

Surface morphology was evaluated using field emission scanning electron microscope (FESEM; Hitachi S-4500, NY, USA) and elemental analysis was carried out using energy dispersive spectroscopy (EDS, Oxford Instruments, MA, USA). The surface roughness was performed on an 840.6 μm X 840.6 μm area using an optical profiler (Nexview, Zygo Corporation, Middlefield, Connecticut, USA). The contact angle measurements were performed by sessile drop method using a contact angle measurement system (VCA optima, AST Products, Inc., MA, USA). A 2 μl distilled water droplet was used for the test and the contact angle between the droplet and the substrate surface was calculated. The mean and SDs were calculated for both surface roughness and contact angle.

### Cell culture

The human bone marrow-derived mesenchymal stromal cell (BMSC) line Saka-T (referred to as Saka cells) was generously provided by Dr. David Roodman (University of Pittsburgh, PA, USA). Cells were cultured in MEM-α supplemented with 10% fetal bovine serum (FBS) and antibiotics/antimycotics (anti-anti) as previously published [18]. Cells were used for experiments when they reached approximately 80–90% confluency.

### Proliferation assay, cellular morphology and quantitative reverse-transcription PCR (qRT-PCR)

Cells were seeded at density of 25x10^3^ and 1x10^6^ to each Ti disc in triplicate wells in 24-well plate for proliferation assay and qRT-PCR, respectively. Cells were allowed to adhere for approximately 30 minutes and 400 μl of media were carefully added from the side of each well followed by incubation at 37°C. After 3 days, cell culture media was removed and 700 μl of DPBS (Fisher Scientific, Waltham, MA, USA) were used to carefully wash cells without disturbing cells. Ti discs were then placed into a new 24-well plate and 400 μl of DPBS were added to each well followed by 100 μl of XTT working solution (Trevigen, Gaithersburg, MD, USA) was added to each well and incubated at 37°C for 3 hours, followed by data acquisition on an Epoch plate reader (BioTek, Winooski, VT, USA).

For qRT-PCR, 1x10^6^ cells were used for each Ti disc in triplicate wells in 24-well plate as indicated above. After 24 hours, cell culture media was aspirated, and cells were lysed using 1 ml TRIzol reagent (Life Technologies, Grand Island, NY, USA). Total RNA was isolated following manufacturer’s protocol. Reverse transcription reactions were conducted using Moloney murine leukemia virus (M-MLV) reverse transcriptase (Promega, Madison, WI, USA) and qRT-PCR was performed using SYBR Green methodologies a ViiA7 real-time PCR instrument (Life Technologies, Grand Island, NY, USA). To quantify gene expression, GAPDH was used as a housekeeping gene and the following primers were used: GAPDH, 5’-CTCGACTTCAACAGCGACA- 3’ (forward) and 5’-GTAGCCAAATTCGTTGTCATACC-3’ (reverse); IL-6, 5’-TCCAAAGATGTAGCCGCCC-3’ (forward) and 5’-CAGTGCCTCTTTGCTGCTTTC-3’ (reverse); GLI2, 5’-CTCCGAGAAGCAAGAAGCCA- 3’ (forward) and 5’-GATGCTGCGGCACTCCTT- 3’ (reverse), CD40L, 5’-AACATCTGTGTTACAGTGGGCT- 3’ (forward) and 5’-AACGGTCAGCTGTTTCCCAT- 3’ (reverse); alkaline phosphatase-1 (ALP-1), 5’-CCTACCAGCTCATGCATAACA-3’ (forward) and 5’-GGCTTTCTCGTCACTCTCATAC-3’ (reverse); collagen A-1 (COLA-1), 5’-CGATGGATTCCAGTTCGAGTATG-3’ (forward) and 5’-CGATGGATTCCAGTTCGAGTATG-3’ (reverse); and osteocalcin (OCN), 5’-CAGGCGCTACCTGTATCAAT-3’ (forward) and 5’-CGATGTGGTCAGCCAACT-3’ (reverse).

Cell morphology was examined using FESEM. After 3 days of culture, samples were removed from culture and fixed with 2% paraformaldehyde/2% glutaraldehyde in 0.1 M phosphate buffer overnight at 4 °C. Samples were rinsed with 0.1 M phosphate buffer (three times, followed by fixation with 2% osmium tetroxide (OsO_4_) for 2 h at room temperature. Samples were then rinsed again with 0.1 M phosphate buffer three times and dehydrated in ethanol series (30, 50, 70, 95, and 100% (three times for 100% ethanol), followed by dehydration using hexamethyldisilane (HDMS; Fisher Scientific, Waltham, MA, USA).

### Statistical analysis

A one-way analysis of variance (ANOVA) was used to analyze data. A *p* value <0.05 was considered significant. Statistical analysis was performed using GraphPad Prism software (San Diego, CA, USA).

## Results

### Anodization and sample characterization

**Fig 1:**
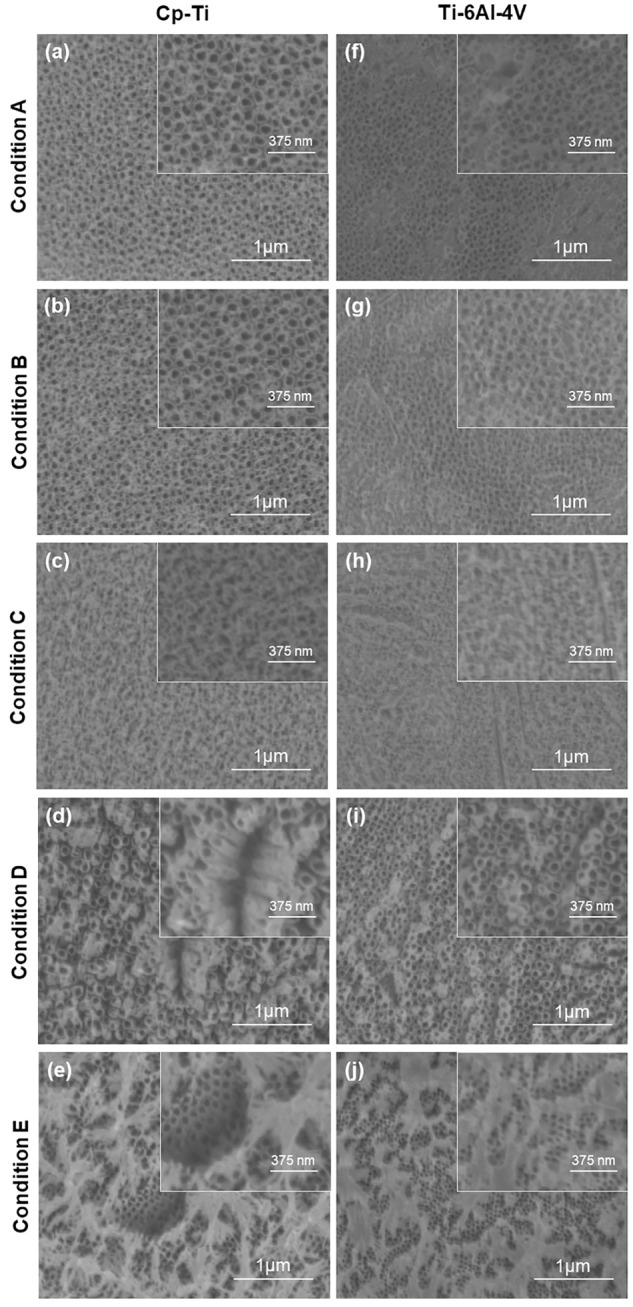
Effect of anodization parameters on surface morphology. Anodization was performed with five different conditions and their effect on nanotubes formation were observed on Cp-Ti (a-e) and Ti-6Al-4V (f-j) using a FESEM. Uniform nanotubes were observed on Ti (a&b) and Ti-6Al-4V (i). Other anodization conditions resulted in nonuniform distribution and/or distortion in structure of nanotubes (c, d, f, g, h) and formation of nanograss (e and j).

Surface morphology of the anodized CP-Ti samples is presented in Fig 1a–1e. Uniform nanotubes with a diameter of ~100 ± 10 nm were generated by using the HF in aqueous electrolyte for 45 min (Fig 1a) and as shown in Fig 1b, increasing the time to 60 min did not affect the morphology. Increasing the voltage to 30 V resulted in formation of random porous structures (Fig 1c). Furthermore, nanotubes with broken structures were formed by having a mixture of NH_4_F and HF in as an electrolyte (Fig 1d). Interestingly, as shown in Fig 1e, changing the electrolyte and having NH_4_F as the only fluoride ion source in ethylene glycol medium resulted in formation of nanograss-like tubular structures. Nanograss are close-packed clusters of nanotubes on top of nanotubes [9]. Similar anodization parameters were used to investigate the role of time, voltage and electrolyte composition on morphology of samples. As illustrated in Fig 1f and 1g, nanotubes were not completely formed in aqueous electrolytes. While nanotubes had a non-uniform structure at lower voltage and anodization time, a randomly porous structure was obtained by increasing time and voltage (Fig 1h). However, uniform nanotubes on Ti-6Al-4V samples with diameter of ~100 ± 15 nm were achieved using a combination of HF and NH_4_F in ethylene glycol (Fig 1i). As shown in Fig 1j, the absence of HF and extended duration time resulted in the formation of nanograss. Taken together, these results indicate that a successful anodization process and uniform nanotube formation depend not only on the anodization parameters, but also on the composition of the Ti substrate.

**Fig 2:**
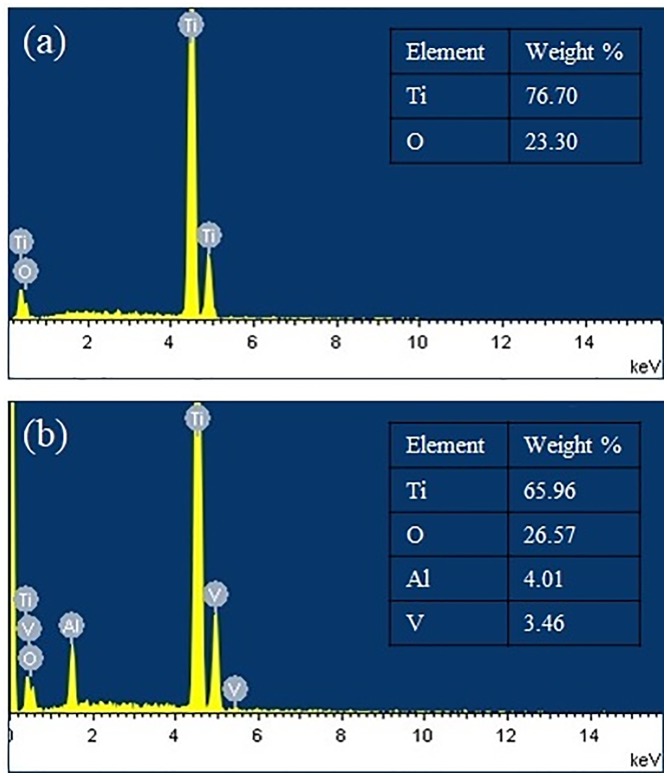
Elemental analysis of Ti substrates. The EDS elemental analysis of (a) Ti-NT and (b) Ti-6Al-4V-NT showing presence of only Ti and O in Ti-NT, while Ti, Al, V and O are found in Ti-6Al-4V.

**Fig 3:**
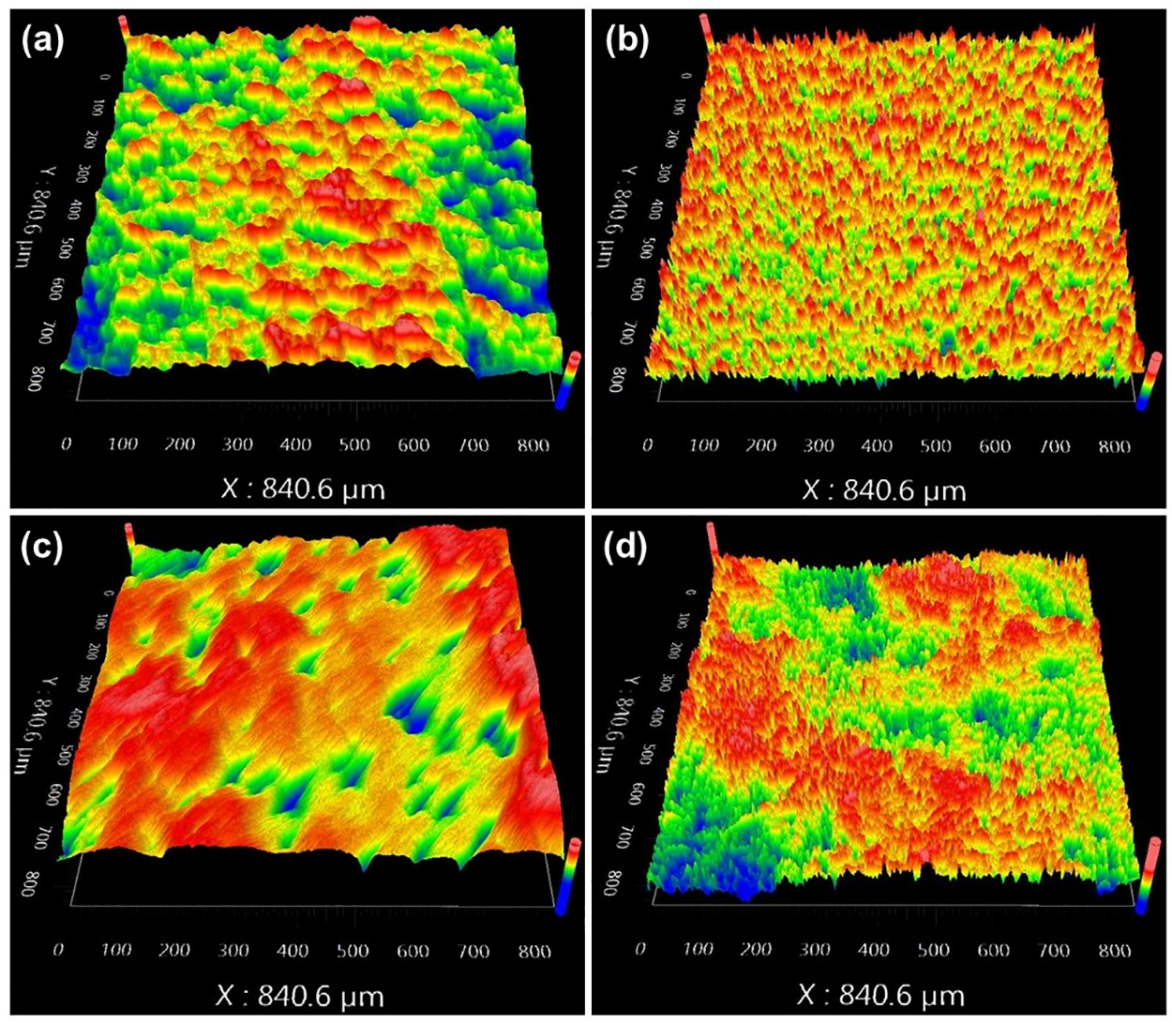
Surface roughness of Ti substrates. Surface roughness of the Ti substrates (a) Ti (b) Ti-NT (c) Ti-6Al-4V (d) Ti-6Al-4V-NT, was determined by an optical profiler. Shows increase in roughness of samples after anodization.

**Fig 4:**
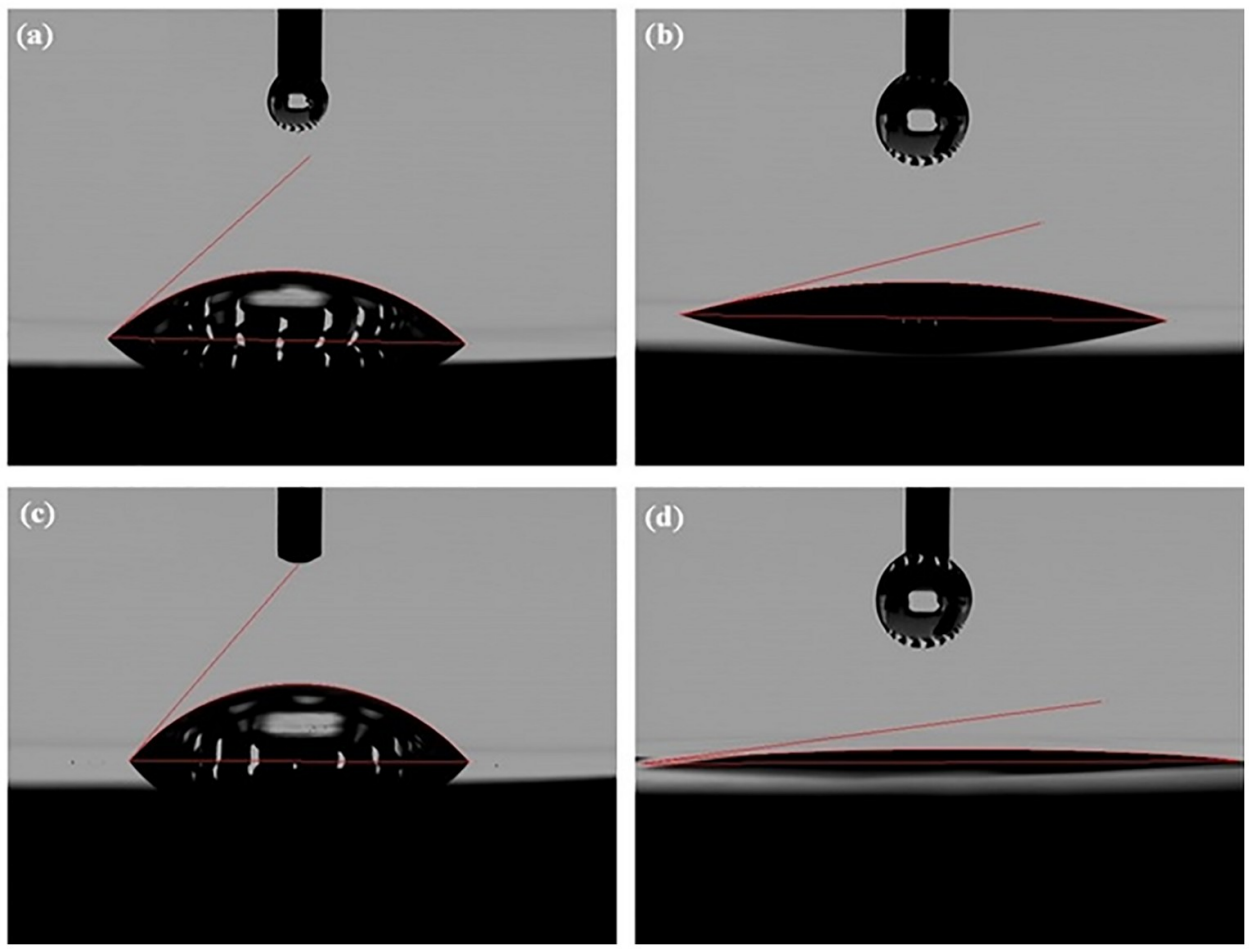
Anodization increases hydrophilicity. Contact angle of (a) Ti (b) Ti-NT (c) Ti-6Al-4V (d) Ti-6Al-4V-NT. Contact angles in anodized samples (b and d) are significantly less that untreated samples showing the effective role of anodization on increasing hydrophilicity.

The optimized anodization conditions that result in well-formed nanotubes on CP-Ti and Ti-6Al-4V were conditions A and D, respectively (referred to as Ti-NT and Ti-6Al-4V -NT, respectively from now on). The surface composition of Ti-NT and Ti-6Al-4V -NT was determined using an EDS detector and the obtained spectrum and elemental analysis are shown in Fig 2. The surface roughness parameters of Ti, Ti-NT, Ti-6Al-4V and Ti-6Al-4V–NT are presented in Table 2 and Fig 3. We found that Ti-NT have a higher surface roughness compared to Ti while no significant change was observed between Ti-6Al-4V and Ti-6Al-4V-NT. Contact angles of samples with water are shown in Fig 4. The contact angles of Ti and Ti-6Al-4V were 44.1°±4 and 48.9°±2, respectively. Formation of nanotubes reduced the contact angles to 20.73°±5 and 14.85°±5 for Ti-NT and Ti-6Al-4V-NT, respectively which suggests enhanced hydrophilicity after anodization.

**Table 2:** Surface roughness parameters of the Ti substrates.

Sample	Sa (nm)	Sq (nm)	Sz (nm)
**Ti**	32±2	39.75±2	342.25±87
**Ti-NT**	196±18	255.9±28	2605.25±113
**Ti-6Al-4V**	36.25±2	46.5±3	419±72
**Ti-6Al-4V-NT**	40.25±6	50.5±8	633±83

### Adhesion of bone marrow stromal cells (BMSCs) on Ti substrates

**Fig 5:**
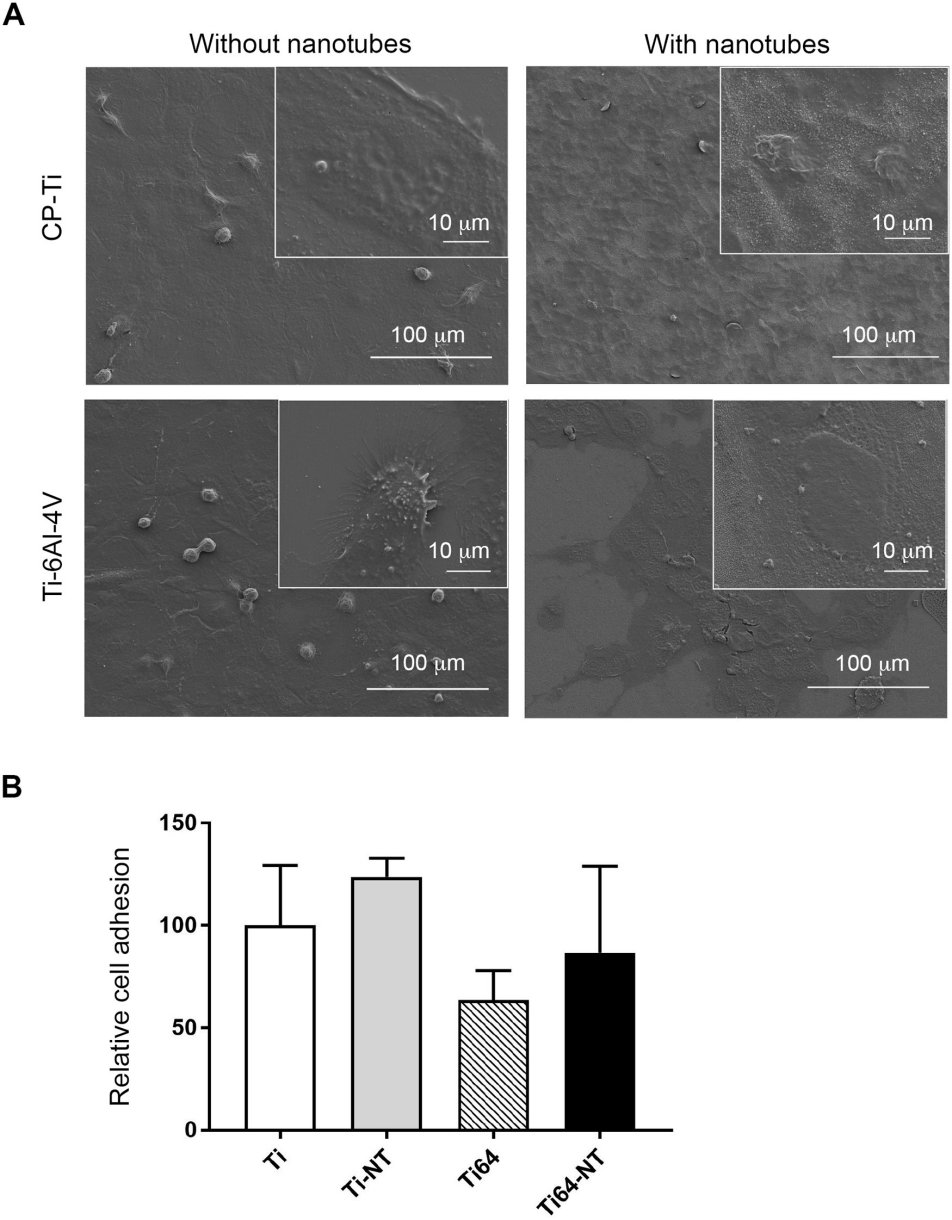
Cellular morphology on Ti substrates. a) Saka cells were grown on Ti discs as indicated in materials and methods followed by examination of cellular morphology using FESEM. b) Saka cells were allowed to adhere to Ti discs for 6 hours followed by investigation of cell adhesion by XTT assay.

The effects of Ti substrate composition and the presence of nanotubes on BMSC attachment and cellular morphology after 3 days of culture were investigated by SEM and the results are shown in Fig 5A. Regardless of composition and surface morphology, BMSCs attached to Ti and Ti-6Al-4V samples and demonstrated well-spread morphologies. We also investigated BMSC adhesion to Ti substrates at earlier times (6 hours following seeding) and assed cell adhesion by XTT assay. We found no differences in cell adhesion between the different substrates compared to adhesion of BMSCs on CP-Ti (p = 0.6056)(Fig 5B).

### Assessment of cell growth, differentiation and inflammatory gene expression on Ti substrates

**Fig 6:**
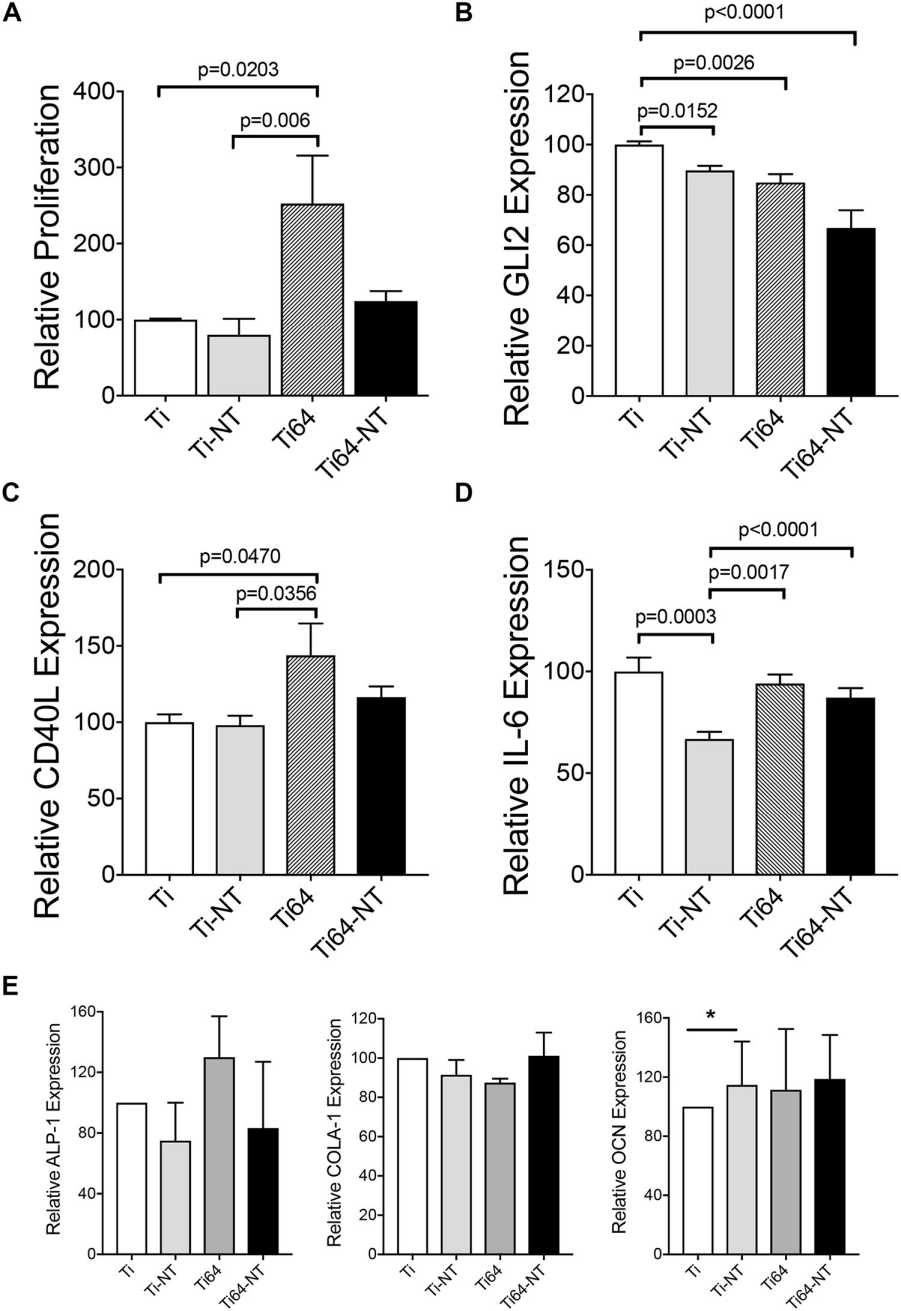
Effect of composition and surface morphology on growth of BMSCs and inflammation. a) Saka cells were allowed to adhere on material as indicated in methods for 3 days followed by XTT assay to determine cell proliferation. qRT-PCR for the inflammatory markers b) GLI2, c) CD40L and d) IL-6. E) Saka cells were allowed to adhere A similar experiment was performed to determine the expression of differentiation markers alkaline phosphatase 1 (ALP-1), Collagen A-1 (COLA-1) and osteocalcin (OCN) on the different substrates by qRT-PCR. Data are presented as averages of 2 independent experiments, each performed in triplicate and the bars represent means ± SEM.

The effect of composition and surface treatment on cell proliferation was investigated using XTT assay and results are presented in Fig 6. As compared to Ti, BMSC proliferation was significantly higher on Ti-6Al-4V (p = 0.0203). However, the incorporation of nanotubes to Ti-6Al-4V (Ti-6Al-4V -NT) reduced the proliferation of BMSC compared with Ti-6Al-4V, suggesting that the incorporation of nanotubes may provide therapeutic efficacy by promoting bone formation [19] but not BMSC growth. We have previously reported that the glioma-associated protein 2 (GLI2) is expressed by BMSCs and modulates inflammatory genes [18,20,21]. Therefore, we examined the expression of GLI2 in BMSCs grown on Ti and Ti-6Al-4V in the presence or absence of nanotubes. We found a decrease in GLI2 expression by BMSCs grown on Ti-NT compared with CP-Ti (Fig 6b) suggesting that downstream inflammatory genes may be reduced. Although Ti-6Al-4V induced BMSC growth, it resulted in a reduction in GLI2 expression (Fig 6b). Furthermore, Ti-6Al-4V-NT had significantly lower GLI2 expression compared with Ti alone. We have previously reported that GLI2 can regulate the expression of CD40 ligand (CD40L) in BMSCs [18]. CD40L expression was increased in BMSCs grown on Ti-6Al-4V but not Ti-6Al-4V -NT (Fig 6c). This pattern of CD40L expression is consistent with the pattern of cell proliferation shown in Fig 6a. We further examined the expression of the pleiotropic cytokine interleukin-6 (IL-6), which is regulated, in part, by GLI2 [20]. Although Ti-6Al-4V induced BMSC proliferation, this did not increase IL-6 expression (Fig 6d). Additionally, Ti-NT, but not Ti-6Al-4V-NT, reduced IL-6 expression. Taken together, these results suggest that the incorporation of NT on Ti-6Al-4V may be therapeutically beneficial by promoting osteoblast attachment as previously reported [22], without inducing BMSC growth, while not altering the inflammatory response required to facilitate healing. Interestingly, BMSCs cultured on CP-Ti and Ti-6Al-4V did not express osteoblast differentiation markers alkaline phosphatase-1 (ALP-1), collagen A-1 (COLA-1) or osteocalcin (OCN) suggesting that the different Ti substrates and the presence of nanotubes did not affect BMSC differentiation (Fig 6e).

## Discussion

TiO_2_ nanotubes are of great interest due to their excellent physical, mechanical and biological properties. Previous studies have reported that nanotubes enhance the protein adsorption and osteoblast cell attachment which improves bioactivity [23,24]. In this study, we fabricated nanotubes on the surface of both CP-Ti and Ti-6Al-4V using experimental different conditions. These conditions were selected based on previous work for different types of titanium alloys [8,9]. We found that formation of nanotubes depends on both anodization parameters and substrate composition. This is in line with the literature where formation of nanotubes was confirmed on IMI834 titanium alloy using H_3_PO_4_+HF electrolyte, whereas no nanotube was found on CP-Ti and Ti-6Al-4V substrates using the same anodization conditions, showing the dependency of the successful anodization on phase composition of Ti and its alloys [6]. Nanotubes formed by anodization are usually referred to as TiO_2_ nanotubes. However, pure TiO_2_ nanotubes are formed only on CP-Ti while the presence of Al and V in Ti-6Al-4V results in formation of Ti-Al-V-O nanotubes. High amounts of Al decrease the dissolution rate, while the presence of V increases the dissolution rate which affect nanotube formation [25]. Similar to Al and V, increase in Zr content in Ti-alloy increases interspace between TiO_2_ nanotubes [26]. However, regardless of Ti composition, the presence of fluoride ions appears crucial for the growth of TiO_2_ nanotubes, as F^-^ helps in the chemical dissolution of the oxide layer. In addition to F^-^, water content and the viscosity of ethylene glycol (EG) alter nanotube formation as they alter the rate of oxidation and diffusion of ions in the electrolyte, respectively [27,28].

The EDS results confirm the presence of oxygen on both Ti-NT and Ti-6Al-4V -NT which was 23.30 and 26.57 wt% respectively while no fluorine was detected (Fig 2). They also confirm the presence of Al and V on Ti-6Al-4V -NT with a percentage of 4.01% and 3.46% respectively (Fig 2). Surface roughness and wettability are the most important factors that influence cell attachment [29,30]. It was reported that the presence nanotubes increases the surface roughness which further improves osseointegration [31]. Our results (Fig 3) are in agreement with the studies with one exception. The presence of nanotubes on Ti increased the surface roughness while no significant change was observed in surface roughness of Ti-6Al-4V with and without nanotubes (Fig 3). This might be due to the presence of porosity in LENS processed Ti-6Al-4V. On the other hand, the results of wettability are in line with the studies reporting the presence of nanotubes reduces the contact angle and increases the hydrophilicity of the surface [32]. Both Ti-NT and Ti-6Al-4V-NT showed improved surface hydrophilicity (Fig 4). Highly hydrophilic surfaces increase cell attachment and improve bioactivity due to enhanced interaction between cells and material [33–35].

We report that in the presence of nanotubes, BMSCs showed well-spread cellular morphology (Fig 5) which may be related to enhanced hydrophilicity in the presence of nanotubes [12]. In addition to morphology, SEM images show the ability of BMSCs to attach and spread on both CP-Ti and Ti-6Al-4V in the presence and absence of nanotubes (Fig 5). Our results also show that the presence of nanotubes reduced proliferation of BMSC which suggests that nanotubes may provide therapeutic efficacy by promoting bone formation as described in earlier *in vivo* studies [19] but not BMSC growth or their differentiation into osteoblasts (Fig 6). Furthermore, when we investigated inflammatory gene expression, we found a reduction in GLI2 expression with Ti-NT and Ti-6Al-4V-NT compared to Ti and Ti-6Al-4V suggesting that downstream inflammatory genes that are regulated by this protein may also be reduced. We previously showed that GLI2 can regulate the expression of CD40 ligand (CD40L) in BMSCs [18]. CD40L is a protein that is expressed on the surface of various cells including stromal cells [18] and plays a role in B-cell activation. Recruitment of B-cells and other immune cells has been shown to promote early bone healing [36]. These results suggest that formation of well-organized nanotubes depend on titanium composition and anodization parameters and Ti-6Al-4V -NT may provide a benefit by maintaining IL-6 expression to allow an initial inflammatory response to mediate healing while not inducing prolonged activation of other immune cells.

### Conclusion

In this study, we investigated the effects of substrate composition, electrolyte, voltage and anodization time on successful nanotube arrangement on Ti substrate and found both substrate composition and anodization parameters effect the formation of nanotubes. We also studied the effects of titanium composition and nanotube presence on their interaction with human BMSCs. For bone tissue engineering applications, the incorporation of NT on Ti-6Al-4V may be therapeutically beneficial due to the ease of manufacturing. Furthermore, this will promote osteoblast attachment [22], without inducing BMSC growth, or altering the inflammatory response required to facilitate healing.

## Supporting information

S1 Table Surface roughness and contact angle measurements. Raw data for measurements of surface roughness and contact angles.
(PDF)

S2 Table XTT assay for Figs 6A and 5B. Raw data from experiments of relative cell proliferation.
(PDF)

S3 Table Gene expression for Fig 6B–6D. Raw data from experiments to determine relative gene expression.
(PDF)

S4 Table Cell differentiation Fig 6E. Raw data from experiments to determine cell differentiation markers by qRT-PCR.
(PDF)
