# Supplementary material for: Bone marrow stromal cells interaction with titanium; Effects of composition and surface modification
Source: PLoS One. 2019 May 22;14(5):e0216087. doi: 10.1371/journal.pone.0216087 (PMC6530826; doi:10.1371/journal.pone.0216087)
Supplement: S1 Table — Raw data for measurements of surface roughness and contact angles. (PDF) [file pone.0216087.s001.pdf]

Surface roughness Sa

Sample	Location	Ti µm	Ti+NT µm	Ti64 µm	Ti64+NT µm
Sample 1	1	0.033	0.18	0.033	0.041
	2	0.03	0.176	0.036	0.036
Sample 2	1	0.03	0.218	0.037	0.05
	2	0.035	0.21	0.039	0.034
		0.032	0.196	0.03625	0.04025
		0.00212132	0.01827567	0.00216506	0.006179604
		32	196	36.25	40.25
		2	18	2	6

Surface roughness Sq

Sample	Location	Ti µm	Ti+NT µm	Ti64 µm	Ti64+NT µm
Sample 1	1	0.041	0.233	0.042	0.052
	2	0.037	0.2226	0.048	0.046
Sample 2	1	0.038	0.288	0.047	0.062
	2	0.043	0.28	0.049	0.042
		0.03975	0.2559	0.0465	0.0505
		0.002384848	0.02848034	0.00269258	0.00753326
		39.75	255.9	46.5	50.5
		2	28	3	8

Surface roughness Sz

Sample	Location	Ti µm	Ti+NT µm	Ti64 µm	Ti64+NT µm
Sample 1	1	0.273	2.542	0.395	0.628
	2	0.243	2.456	0.54	0.632
Sample 2	1	0.402	2.746	0.349	0.753
	2	0.451	2.677	0.392	0.519
		0.34225	2.60525	0.419	0.633
		0.086664223	0.11317547	0.07219072	0.082797947
		342.25	2605.25	419	633
		87	113	72	83

Contact angle measurements

Sample	Data	Ti	Ti+NT	Ti64	Ti64+NT
Sample 1	1	42.7	15.2	45.4	15.8
	2	50.15	24.55	48.7	22.05
Sample 2	1	41.05	17.1	50.7	7.4
	2	42.65	26.1	50.85	14.15
		44.1375	20.7375	48.9125	14.85
		4	5	2	5
