# Supplementary material for: Bone marrow stromal cells interaction with titanium; Effects of composition and surface modification
Source: PLoS One. 2019 May 22;14(5):e0216087. doi: 10.1371/journal.pone.0216087 (PMC6530826; doi:10.1371/journal.pone.0216087)
Supplement: S2 Table — Raw data from experiments of relative cell proliferation. (PDF) [file pone.0216087.s002.pdf]

		Ti	Ti-NT	Ti64	Ti64+NT
day 3 XTT	Exp.1	102		420	
		98		547	
	Exp.2	96	16	137	163
		104	75	230	107
			146	186	
	Exp.3	97	96	129	106
		103	67	120	121

		Ti	Ti-NT	Ti64	Ti64+NT
adhesion	6 hours	107	139	78	50
		46	125	49	39
		147	107		171
