# Supplementary material for: Bone marrow stromal cells interaction with titanium; Effects of composition and surface modification
Source: PLoS One. 2019 May 22;14(5):e0216087. doi: 10.1371/journal.pone.0216087 (PMC6530826; doi:10.1371/journal.pone.0216087)
Supplement: S3 Table — Raw data from experiments to determine relative gene expression. (PDF) [file pone.0216087.s003.pdf]

		Relative expression			
		Ti	Ti+NT	Ti64	Ti64+NT
GLI2	Exp.1	102	94	70	14
		92	93	67	15
		111	91	65	14
		102	80	73	44
		105	79	70	45
		101	83	69	45
		97	84	96	20
		98	84	85	21
		93	85	92	19
	Exp.2	110	65	77	78
		107	75	68	77
		94	101	61	87
		94	91	64	87
		98	90	103	80
		98	89	98	77
	Exp.3	92	99	112	88
		96	97	99	93
		92	99	104	89
		106	102	93	87
		105	94	84	88
		103	89	86	92
		103	95	102	112
		94	94	105	125
		108	100	97	107

		Relative expression			
		Ti	Ti+NT	Ti64	Ti64+NT
IL-6	Exp.1	99	81	83	92
		93	87	80	90
		89	74	78	110
		89	80	75	95
		109	73	116	108
		121	76	112	107
	Exp.2	64	48	88	61
		69	49	89	59
		65	48	89	53
		94	55	79	78
		94	53	83	81
		88	57	82	77
		151	76	115	99
		138	72	122	98
		136	74	120	99

		Relative expression			
		Ti	Ti+NT	Ti64	Ti64+NT
CD40L	Exp.1	109	108	76	177
		88	116	72	161
		116	98	64	187
		98	101	160	160
		99	108	177	106
		94	109	141	104
		111	125	150	103
		93	84	150	122
		93	85	128	110
	Exp.2	135	74	320	126
		142	60	453	150
		69	198	395	77
		66	134	133	87
		100	53	101	131
		88	56	99	123
	Exp.3	69	80	75	74
		65	74	87	84
		65	81	69	93
		92	90	76	72
		92	97	66	74
		93	109	73	70
		136	106	118	140
		142	101	133	133
		145	110	136	131
