# Supplementary material for: Bone marrow stromal cells interaction with titanium; Effects of composition and surface modification
Source: PLoS One. 2019 May 22;14(5):e0216087. doi: 10.1371/journal.pone.0216087 (PMC6530826; doi:10.1371/journal.pone.0216087)
Supplement: S4 Table — Raw data from experiments to determine cell differentiation markers by qRT-PCR. (PDF) [file pone.0216087.s004.pdf]

		Ti	Ti+NT	Ti64	Ti64+NT
ALP-1	EXP.1	78	86	123	136
		122	114	191	118
	EXP.2	100	51	91	44
		100	49	115	35

		Ti	Ti+NT	Ti64	Ti64+NT
ColA-1	EXP.1	99	82	90	91
		101	86	89	88
	EXP.2	89	102	89	118
		111	96	82	108

		Ti	Ti+NT	Ti64	Ti64+NT
OCN	EXP.1	100	87	67	95
		100	84	74	83
	EXP.2	101	146	152	156
		99	142	153	141
